# Microenvironmental Determinants of Breast Cancer Metastasis: Focus on the Crucial Interplay Between Estrogen and Insulin/Insulin-Like Growth Factor Signaling

**DOI:** 10.3389/fcell.2020.608412

**Published:** 2020-12-08

**Authors:** Veronica Vella, Ernestina Marianna De Francesco, Rosamaria Lappano, Maria Grazia Muoio, Livia Manzella, Marcello Maggiolini, Antonino Belfiore

**Affiliations:** ^1^Endocrinology, Department of Clinical and Experimental Medicine, University of Catania, Garibaldi-Nesima Hospital, Catania, Italy; ^2^Department of Pharmacy, Health and Nutritional Sciences, University of Calabria, Rende, Italy; ^3^Center of Experimental Oncology and Hematology, Azienda Ospedaliera Universitaria (A.O.U.) Policlinico Vittorio Emanuele, Catania, Italy; ^4^Department of Clinical and Experimental Medicine, University of Catania, Catania, Italy

**Keywords:** breast cancer, metastasis, estrogen receptor, GPER, insulin/IGF signaling, tumor microenvionment, targeted therapies

## Abstract

The development and progression of the great majority of breast cancers (BCs) are mainly dependent on the biological action elicited by estrogens through the classical estrogen receptor (ER), as well as the alternate receptor named G-protein–coupled estrogen receptor (GPER). In addition to estrogens, other hormones and growth factors, including the insulin and insulin-like growth factor system (IIGFs), play a role in BC. IIGFs cooperates with estrogen signaling to generate a multilevel cross-communication that ultimately facilitates the transition toward aggressive and life-threatening BC phenotypes. In this regard, the majority of BC deaths are correlated with the formation of metastatic lesions at distant sites. A thorough scrutiny of the biological and biochemical events orchestrating metastasis formation and dissemination has shown that virtually all cell types within the tumor microenvironment work closely with BC cells to seed cancerous units at distant sites. By establishing an intricate scheme of paracrine interactions that lead to the expression of genes involved in metastasis initiation, progression, and virulence, the cross-talk between BC cells and the surrounding microenvironmental components does dictate tumor fate and patients’ prognosis. Following (i) a description of the main microenvironmental events prompting BC metastases and (ii) a concise overview of estrogen and the IIGFs signaling and their major regulatory functions in BC, here we provide a comprehensive analysis of the most recent findings on the role of these transduction pathways toward metastatic dissemination. In particular, we focused our attention on the main microenvironmental targets of the estrogen-IIGFs interplay, and we recapitulated relevant molecular nodes that orientate shared biological responses fostering the metastatic program. On the basis of available studies, we propose that a functional cross-talk between estrogens and IIGFs, by affecting the BC microenvironment, may contribute to the metastatic process and may be regarded as a novel target for combination therapies aimed at preventing the metastatic evolution.

## Introduction

Breast cancer (BC) is the most common tumor in women and the second cause of cancer-related death worldwide (DeSantis et al., 2019). The metastatic evolution, which occurs in nearly 50% of BC patients, seriously thwarts the clinical management of the disease, thereby representing one of the main determinants of BC mortality (DeSantis et al., 2019). Consequently, enormous research effort is currently focused on a better understanding of the multiple molecular and biological factors facilitating the formation and spread of metastases. In this vein, gene signatures specifically discriminating between metastatic and non-metastatic tumors have been identified (Ramaswamy et al., 2003), allowing to postulate that the metastatic propensity is established in the early stages of oncogenesis by three major classes of genes: (i) genes controlling the metastasis initiation, (ii) genes controlling the metastasis progression, and (iii) genes controlling the metastasis virulence (Nguyen and Massagué, 2007). In addition, it is now recognized that most of these genes activated in cancer cells coopt microenvironmental signals to prompt the metastatic process in diverse tumor types, including BC. Indeed, the acquisition of metastatic features requires a complex and coordinated interaction between the epithelial BC cells and the surrounding tumor microenvironment, which is characterized by cellular (stromal fibroblasts, adipocytes, cancer stem cells (CSCs), and endothelial and immune cells) and non-cellular [growth factors and hormones, extracellular matrix (ECM) molecules, cytokines, and low oxygen] components that actively cooperate toward the metastatic landscape (Hanahan and Coussens, 2012). In this context, it should be mentioned that certain metabolic conditions associated with dysfunctional hormonal status, such as obesity and type 2 diabetes, may contribute to metastasis formation in BC, as suggested by epidemiological evidence indicating an elevated risk of metastasis in diabetic and obese patients (Park et al., 2017; Harding et al., 2020). Likewise, worse prognostic parameters have been detected in this subpopulation of BC patients (Schrauder et al., 2011; Zhao and Ren, 2016). Notwithstanding the aforementioned epidemiological correlations, the molecular mechanisms underlying the high risk and poor outcome of obese and diabetic BC patients are complex and multifactorial. First, adipose tissue does contribute to the local production of estrogens, which exert a potent stimulatory action on cancer cells binding to the classical estrogen receptor (ER), as well as the alternate G-protein–coupled estrogen receptor (GPER) (Barton et al., 2018). In addition, obesity facilitates the establishment of hyperinsulinemia and insulin resistance, thereby determining an unopposed activation of the insulin receptor (IR) and the insulin-like growth factor receptor (IGF-1R) (Lewitt et al., 2014), which are part of the complex insulin/IGF system (IIGFs). IIGFs comprises insulin, IGF-1, IGF-2 (IGFs) and cognate receptors (IR, IGF-1R, IR/IGF-1R hybrids, and IGF-2R also known as the mannose 6-phosphate receptor) and six IGF-binding proteins (IGF-BP1-6) (Frasca et al., 2008). IIGFs is deeply deregulated in diverse type of tumors, including BC, and it has been implicated in the acquisition of the metastatic potential (Frasca et al., 2008; Vigneri et al., 2015; Manzella et al., 2019). Noteworthy, both IIGFs and estrogen signaling promote paracrine responses that endow cross-communications within the diverse components of the breast tumor microenvironment toward metastatic progression. In addition, complex networks of molecular and functional connections between these signaling systems appear to elicit a relevant role in BC metastasis. Herein, we first provide a comprehensive analysis of the most significant components of the tumor microenvironment involved in the activation of metastatic programs in BC. Next, we emphasize the molecular and functional interplay between estrogen and IIGFs signaling in activating BC microenvironment toward the acquisition of a metastatic phenotype. Finally, we propose that targeting the dysfunctional interactions between the IIGFs and the estrogen pathways may represent a promising tool in comprehensive therapeutic approaches aimed at halting the aberrant microenvironment in the metastatic BC.

## Microenvironmental Players Involved in BC Metastasis

Despite being considered a biologically inefficient process, as only few of the cancer cells released in the bloodstream actually develop secondary tumors, metastases remain one of the most intriguing and investigated aspects of tumor biology for their huge impact on prognosis. Likewise, the vast majority of BC-related deaths are due to metastases, which target mainly the bone (50–75%), lung (17%), brain (16%), and liver (6%) (Wei and Siegal, 2017). BC cells can escape the primary tumor, sneaking into the circulatory system and reaching distant sites where the neoplastic cells can either form a novel tumor mass straight after or enter a dormant state that can end up in disease relapse. Accordingly, the formation of overt secondary tumors can occur even many years after the diagnosis of the primary disease, as tumor cells disseminated at secondary sites may remain indolent for protracted period of times, until systemic and local factors cooperate toward the waken-up of dormant tumors. The macroenvironmental and microenvironmental mechanisms regulating cancer cell detachment from primary site and colonization at secondary target tissues, as well as entry and exit from dormancy, are likely to determine the fate of incipient tumors and therefore the prognosis of patients. In this paragraph, we provide an overview of the main microenvironmental players involved in BC metastasis, in order to provide a propaedeutic outline for depicting the cooperation of estrogen and IIGF signaling in triggering metastasis dissemination. For descriptive purposes, the aforementioned players will be categorized according to their role in (i) metastasis initiation, (ii) metastasis progression, and (iii) metastasis virulence.

### Metastasis Initiation

Metastasis initiation refers to the complex coordination of the biological processes determining tumor outgrowth and angiogenesis, thereby prompting cancer cell entry into the bloodstream. A better understanding of the microenvironmental mechanisms regulating the expression of genes involved in metastasis initiation in BC is pivotal to deciphering the role of estrogenic and IIGFs signaling in the early stages of metastatic switch. In BC, the initiation of metastasis appears to be abundantly regulated by microenvironmental events that promote epithelial-to-mesenchymal transition (EMT), the formation of CSCs, the activation of neoangiogenesis, and the instigation of local invasion.

EMT occurs when epithelial cells are reprogrammed to acquire mesenchymal traits, endowing BC cells with increased detachment propensity, enhanced motility, and invasive capability, as well as augmented intravasation capacity (Polyak and Weinberg, 2009). Clearly, EMT entails a profound change in cytoskeleton organization and a marked inclination to loosen cell–cell junctions that disrupt the contiguity of the epithelium and facilitate the breaching of basement membrane. A number of environmental clues originating from diverse cell types within the tumor milieu may activate EMT programs in BC. The most important regulatory factors in EMT are hormones, growth factors [IGFs, hepatocyte growth factor (HGF), epidermal growth factor [EGF], platelet-derived growth factor [PDGF], transforming growth factor β (TGF-β)], and cytokines/chemokines (Polyak and Weinberg, 2009). In addition, developmental signaling pathways (Wnt, Notch, and Sonic hedgehog), ECM components (collagen, hyaluronic acid, integrins), and local hypoxia may contribute to the modulation of EMT (Polyak and Weinberg, 2009).

These stimuli converge on several EMT-inducing transcription factors such as Snail, Slug, Zeb1, Zeb2, Twist, FoxC2, and Goosecoid (Polyak and Weinberg, 2009), with the ultimate aim to repress CDH1 (E-cadherin) transcription, thereby reducing epithelial differentiation and promoting a mesenchymal phenotype. It is worth recalling the enormous heterogeneity of microenvironmental cell types involved in the production of breast EMT-inducing molecules. For instance, stromal cancer-associated fibroblasts (CAFs) expressing Snail1 are associated with a high degree of desmoplastic areas with anisotropic fibers, together with lymph node involvement and worse prognosis in infiltrating BC (Stanisavljevic et al., 2015). Likewise, Snail-1 depletion in CAFs hampered their paracrine activity toward metastatic invasion, as supported by animal models of BC co-xenografted with BC cells and Snail1-deficient CAFs (Alba-Castellón et al., 2016). Together with fibrous stroma, also adipose stroma is involved in BC EMT toward the acquisition of metastatic potential. In this regard, it has been shown that when cocultured with adipocytes, BC cells may acquire EMT-like phenotypic changes associated with Twist-1 activation and higher migratory and invasive capability (Lee et al., 2015). Extending these findings, transcription factors classically associated with EMT programs have been shown to impact also other aspects of BC progression, including inflammation and antitumor immunity. This is the case for the transcription factor ZEB (zinc finger E-box–binding protein 1), whose global transcriptional regulation profile has been investigated by chromatin immunoprecipitation and RNA sequencing, followed by gene set enrichment analysis of ZEB1-bound genes in BC cells. Using this approach, the authors identified a ZEB1-regulated inflammatory phenotype associated with the production of cytokines classically related with poor prognosis and metastasis, including interleukin 6 (IL-6) and IL-8 (Katsura et al., 2017). Of note, in EMT-activated BC cells, the immune checkpoint ligand PD-L1 was shown to be up-regulated in a Zeb-1–dependent manner (Noman et al., 2017), reinforcing the evidence that EMT-associated gene signatures correlate with increased inflammatory immune cell infiltration toward BC aggressiveness (Mak et al., 2016). It should be mentioned that EMT also serves as a reprogramming tool through which cancer cells acquire stemness features correlated with enhanced metastatic capability (Mani et al., 2008). According to the CSC hypothesis, a rare subpopulation of stem-like cells with tumorigenic, self-renewal and differentiation properties may generate all cell types within the tumor bulk (De Francesco et al., 2018b). Furthermore, metastatic proficiency is strictly linked to the abundance of cancer cells with stem features (Charafe-Jauffret et al., 2009). In cells undergoing EMT, mammosphere formation, used as readout for CSCs activity, is 10-fold more efficient, thereby corroborating the idea that the EMT process may serve as a source of CSCs (Mani et al., 2008). In this context, the adaptive response gene ATF3 has been proposed to integrate stromal signals coming from the tumor microenvironment with the acquisition of combined EMT/CSC features. More specifically ATF3, which is regulated by a number of extracellular signals including TGF-β, tumor necrosis factor α (TNF-α), and IL-1β, may promote morphological and molecular changes consistent with the activation of EMT, the increase of the CD24low–CD44high cells, the formation of mammospheres, the activation of motility programs, and breast tumorigenesis *in vivo* (Yin et al., 2010).

Transendothelial migration (TEM) precedes the dissemination of cancer cells in the circulation, thereby permitting intravasation. As a pivotal step in metastasis initiation, TEM entails a number of microenvironmental cellular and non-cellular actors. Indeed, endothelial cells, vessel-associated macrophages (VAMs) and tumor-associated macrophages (TAMs) play a key role in BC cell intravasation. For instance, VAMs secrete chemoattractant molecules to recruit cancer cells at the vessel interface, whereas BC cells themselves secrete colony-stimulating factor to attract macrophages in an auto-amplifying paracrine loop (Goswami et al., 2005). Moreover, macrophages-derived TNF-α induces the retraction of endothelial cells and their apoptosis, thus rendering vessels more loose and permeable for cancer cells invasion (Zervantonakis et al., 2012). Interesting evidences indicate that diverse signals from stromal CAFs led by TGF-β, PDGF, CXCL12/CXCR4, vascular endothelial growth factor (VEGF), and matrix metalloproteinases (MMPs) can directly drive the process of intravasation through multiple mechanisms as ECM remodeling, enhanced vessel permeability, and aberrant angiogenesis (Guo and Deng, 2018).

### Metastasis Progression

The reciprocal interaction between estrogen and IIGFs signaling in BC microenvironment facilitates metastasis progression, which refers to the multiple events occurring both in the primary tumors and at metastatic sites, immediately after intravasated cancer cells enter the circulation and reach target organs. Having gained access to lymphatic vessels or capillaries, circulating BC cells disperse in the bloodstream in various directions before their extravasation at secondary site, an event that seems to be organ-specific and facilitated by numerous players like components of the TME [mesenchymal stromal cells (MSCs), CAFs, TAMs], circulating cancer cells, and extravasation factors. By using a murine BC model of lung metastasis, Yu and collaborators found that MSCs maintain an inhibitory tone on lung metastasis formation through the release of the inflammatory chemokine CXCL12 and the up-regulation of the cognate receptor CXCR7 in BC cells (Yu et al., 2017). However, this effect is reversed in the presence of TGF-β, thus indicating that the prometastatic effect of MSCs depends on the simultaneous activation of inflammatory pathways like TGF-β, which is known to be activated in CAFs (Yu et al., 2017). The rapid outgrowth and expansion of the neoplastic mass generates intratumor hypoxia, which activates compensatory biological responses mediated by the transcription factors HIF (hypoxia-inducible factors) 1 and 2 (Semenza, 2012). HIF-mediated gene transcription occurs at the primary tumor, at the premetastatic niche, and ideally in all the cellular components of the TME, with the ending result of boosting the formation of metastasis (Semenza, 2012). In BC, HIF triggers the production of angiogenic factors such as VEGF to support intravasation and extravasation (Semenza, 2012). Loss of HIF-1 in triple-negative BCs (TNBCs) was associated with decreased lung metastasis through the inhibition of L1 cell adhesion molecule, which mediates BC cells’ physical interactions with endothelial cells at the pulmonary district (Zhang et al., 2012). Of note, HIF mediates the activation of signaling systems required for BC invasion like the HGF/MET pathway and the RhoA/Rock signaling (Semenza, 2012). Ideally contributing to all the steps necessary for metastasis formation and dissemination, gene transcription programs dependent on HIF activation pave the way for extravasation and invasion also by triggering deep transformations of ECM. These responses require the up-regulation of lysyl oxidase enzymes (LOX, LOXL2, and LOXL4), which are produced by hypoxic BC cells released in the bloodstream and accumulated at premetastatic niche, where they enable the remodeling of collagen and other ECM molecules toward the intravasation of circulating BC cells (Schito and Semenza, 2016). Interestingly, certain ECM molecules such as hyaluronan not only enable tumor stroma with mechanical properties facilitating BC cell motility, but also provide CAFs with enhanced migratory capability leading to the metastasis progression (McCarthy et al., 2018). Indeed, CAFs can be found at the primary and the metastatic stroma, as well as in the circulatory system. Circulating CAFs (cCAFs) can be detected individually or in CAFs clusters, as well as in heterotypic clusters with circulating tumor cells (CTCs). It has been suggested that cCAFs generate a suitable microenvironmental niche for metastasis seeding and growth together with the escape from immune surveillance (Duda et al., 2010). In support of this hypothesis, CAFs have been detected in premetastatic niches prior to the appearance of cancer cells. Extending these findings, Ao and collaborators detected cCAFs in almost 90% of patients with metastatic BC, whereas these cell types were detected in nearly the 20% of patients with localized disease and were absent in samples from healthy donors. These observations indicate that cCAFs may serve as a tool to track and perhaps anticipate the detection of CTCs (Ao et al., 2015). CTCs, which are found as single cells or as clusters (tumor emboli), are considered as precursors of metastatic colonies. Their biology and behavior strictly depend on the tumor of origin, as well as on microenvironmental factors. For instance, a metastasis-competent subset of clustered CTCs from BC patients oligoclonally derive from primary tumor cells and are held together by plakoglobin-mediated intercellular adhesion (Aceto et al., 2014). Interestingly, elevated expression of plakoglobin in BC samples correlates with worse prognostic index, including worse distant metastasis–free survival, thereby reinforcing the role of CTCs and related factors in metastasis formation (Goto et al., 2017). It has become increasingly recognized that TAMs contribute to the acquisition of malignant features in BC, through multiple mechanisms, including the formation and dissemination of metastasis. Indeed, TAMs contribute to BC cell migration and invasion, boost lymphangiogenesis and angiogenesis, participate in the formation of the metastatic niche and maintain a cross-communication with BC cells to support disease progression (Williams et al., 2016). Chemoattractant factors released by TAMs trigger tumor cells intravasation and their travel at distant sites such as lung and bone (Williams et al., 2016). Furthermore, TAMs secrete a number of proangiogenic mediators including EGF, PDGF, MIF, TNF-α, TGF-β, IL-8 and IL-1β, CCL2, and CXCL8 (Williams et al., 2016). Interestingly, intravasation of BC cells facilitated by TAMs can occur also in absence of local angiogenesis, as evidenced by multiphoton microscopy in animal models of BC (Williams et al., 2016). It is been largely demonstrated that paracrine signals between TAMs and BC cells establish positive feedback loops conducive to disease progression. In particular, EGF secreted specifically by TAMs but not by BC cells derived from primary tumors was shown to promote cell invasion (O’Sullivan et al., 1993) and the expression of CFS-1 in BC cells. Then, CSF-1 secreted by BC cells induced the production of EGF by TAMs (Goswami et al., 2005). The pharmacological manipulation of this paracrine cycle by inhibition of either EGFR or CSF-1R was sufficient to dampen BC cell migration and invasion (Goswami et al., 2005). On the basis of the above considerations, it is evident that the BC microenvironment at the metastatic site is profoundly different from that surrounding the primary tumor. Understanding these molecular and biological differences may represent a useful tool to manipulate the tumor microenvironment in order to control the metastatic progression.

### Metastasis Virulence

A number of estrogen and IIGF-regulated genes control the so-called metastasis virulence, which refers to the events that contribute to the metastatic colonization. These multifaceted responses bestow biological advantages to the secondary rather than the primary tumor, facilitating the establishment of macrometastases once locally aggressive micrometastasis have been formed. Clearly, the mechanisms regulating these responses are particularly influenced by the organospecific tropism of metastatic BC cells; however, general dynamic mechanisms governing metastasis virulence can be described. First, BC cells that successfully reach secondary sites are subjected to a mesenchymal–epithelial transition, which restores the epithelial phenotype. Afterward, neoplastic cells within the metastatic niche activate paracrine signaling that allow cell survival, resistance to apoptosis, evasion from immune surveillance, and colonization. Bone represents the main site for BC metastasis, particularly in the luminal subtypes of BC (Wei and Siegal, 2017). Metastatic BC cells hamper bone remodeling, promote bone degradation, and activate osteomimicry processes that facilitate the formation of macrometastasis (Awolaran et al., 2016). The initial trigger is represented by factors released by BC cells in the bone, including osteopontin (OPN), parathyroid hormone-related peptide (PTHrP), heparanase, IL-1, IL-6, and prostaglandin E2. These mediators contribute to the instigation of osteolytic processes by RANKL–RANK signaling. As a consequence of osteoclasts activation, bone is degraded through the involvement of cathepsin-K, MMP-9, and MMP-13. Growth factors stored in the bone matrix (TGF-β, IGF-1) are immediately released and in turn stimulate BC cells to secrete additional PTHrP in a vicious cycle (Waning and Guise, 2014). As it concerns brain metastasis from BC, their ability to adapt to the specific brain microenvironment is highlighted by the evidence that novel neurovascular units constituted by metastatic cells, together with microvascular cells, astrocytes, and neurons are immediately organized in the metastatic niche, where neoplastic cells may acquire a metabolic phenotype similar to the ones of resident cells (Neman et al., 2014). Very likely, this strict multicellular cooperation guarantees a better control on the brain blood barrier, thereby facilitating the access of additional CTCs, as well as an easy entry gate for nutrients. Interestingly, brain metastatic cells can activate adjacent astrocytic and glial cells that in turn secrete a number of tumor-stimulating cytokines, including IL-6, interferon γ (IFN-γ), tumor necrosis factor-α (TNF-α), TGF-β, IGF-1, and PDGF-1 (Wang et al., 2013), thereby supporting the role of the metastatic microenvironment in the evolvement of the secondary disease. In order to survive and colonize the hostile lung environment, BC metastases enact a deep remodeling of the premetastatic niche through the establishment of paracrine responses at the interface between host cells and cancer cells. For instance, BC cells exhibiting a preferential tropism for the lung fuse with lung fibroblasts and release their exosomes toward the production of proinflammatory S100 proteins that facilitate the survival of metastatic cells (Hoshino et al., 2015). Additionally, the mobilization of bone marrow–derived cells initiated by the HIF/LOX pathway in hypoxic BC cells triggers ECM remodeling in the lung and facilitates the systemic instigation of indolent cancer cells through the secretion of OPN. Interestingly, Ye et al. (2015) have unveiled the ability of an inflammatory microenvironment to impact on metastasis formation at the lung. More specifically, using a mouse model of BC, the authors found that a TGF-β–driven inflammatory signature drives the secretion of cytokines involved in the formation of the premetastatic niche such as S100A8, S100A9, Angpt2, and VEGF. Last, a metastasis-favorable microenvironment has been hypothesized for liver, where larger BC metastasis can be found compared to the lung. Along with fibroblasts and TAMs, liver-specific cellular components such as Kupffer cells, liver sinusoidal endothelial cells, and hepatic stellate cells cooperate toward the establishment of metastasis (Ma R. et al., 2015). Of note, liver metastases from BC cells show a peculiar metabolic profile compared to bone and lung metastases. The reduction of mitochondrial metabolism and the increased rate of conversion of pyruvate into lactate by PDK1 may suggest a specific metabolic adaptation to lack of nutrients and hypoxia. Likewise, PDK1 is recognized as one of the most important regulators of liver metastasis in BC (Dupuy et al., 2015).

## Estrogen and IIGFs Signaling in BC

**FIGURE 1:**
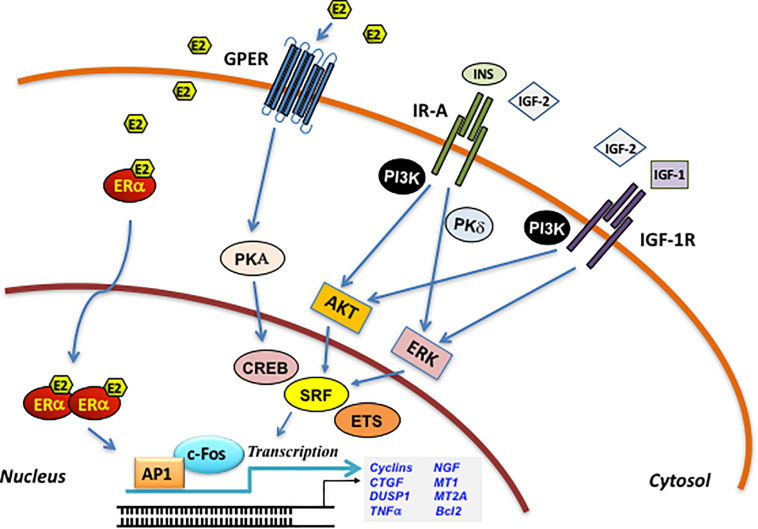
Schematic illustration of the ERα/GPER and IIGFs cross-talk. Insulin, IGF-2, and IGF-1 bind to their specific receptors and stimulate rapid signals converging to the activation of PI3K, MAPK, and PKδ networks. These pathways, in turn, trigger the activation of transcription factors including CREB, SRF, and ETS, which favor c-fos induction and its recruitment to the AP-1 site. ERα/GPER activation by E2, through the activation of various intermediates, cross-talks with the IIGFs leading to enhanced mitogenic signals. PKA, protein kinase A; PKCδ, protein kinase C, δ isoform; MAPK, mitogen-activated protein kinases; PI3K, phosphatidyl-inositol-3-kinases; ERK, extracellular signal-regulated kinases; AKT, protein kinase B; CREB, cAMP-response element-binding protein; ETS, E26 transformation specific; SRF, serum response factor; c-fos, FBJ murine osteosarcoma virus; AP-1, activator protein-1; CTGF, connective tissue growth factor; DUSP1, dual specificity protein phosphatase 1; TNF-α, tumor necrosis factor α; NGF, nerve growth factor; MT1, metallothionein 1; MT2A, metallothionein 2A; Bcl2, B-cell lymphoma 2.

Having described the main biological events and molecular mediators orchestrating the microenvironmental responses involved in BC metastasis, in this paragraph we provide a brief but sound overview of the basic signaling mechanisms mediated by estrogen and IIGFs in BC (Figure 1). Despite the description of estrogen and IIGFs pathway in epithelial BC cells goes beyond the purpose of this review, a concise sketch of the mode of action of these transduction pathways is required to understand how estrogen and IIGFs signaling work together in landscaping BC microenvironment toward metastasis propagation.

### Estrogen Signaling

Estrogenic signaling facilitates the establishment of BC metastasis by activating stimulatory responses that impact the initiation, progression, and virulence of metastatic genes. Most of these genes are transcriptionally regulated by the ERα, which is expressed in approximately 70% of breast tumors identifying estrogens as master regulators of breast malignant development (Katzenellenbogen and Frasor, 2004; Yager and Davidson, 2006; Kumar et al., 2011; Rondón-Lagos et al., 2016). Consequently, ERα is a main target of the current endocrine approaches in ERα-positive BCs (Howell et al., 2007). Estrogen-mediated gene transcription occurs through multiple independent and sometimes cooperating mechanisms that may lead to relevant biological responses. Unliganded ERα is principally located in the cytoplasm; however, upon ligand exposure, it dissociates from the heat shock proteins, dimerizes, and shuttles to the nuclear compartment (Stenoien et al., 2001). Then, ERα acts as a transcription factor binding to the estrogen-responsive elements (EREs) located on the promoter regions of target genes (Stenoien et al., 2001). Ligand-activated ERα may also regulate the transcription of genes in an ERE-independent manner through the interaction with other factors (McDonnell and Norris, 2002; Björnström and Sjöberg, 2005). For instance, interacting with c-fos and c-jun proteins at the AP-1–binding sites, ERα may regulate the transcription of genes as IGF-1 (Umayahara et al., 1994), collagenase (Webb et al., 1995), and cyclin D1 (Sabbah et al., 1999). In addition, ERα may contribute to rapid responses to estrogens by interacting with scaffold proteins such as caveolin-1 or signaling molecules, namely, G proteins, Src kinase, and Shc (Migliaccio et al., 1996; Razandi et al., 1999, 2002; Wyckoff et al., 2001; Song et al., 2002; Auricchio et al., 2008; Levin and Pietras, 2008; Levin, 2009), and activate diverse extranuclear signaling cascades, such as Src, adenylyl cyclase, mitogen-activated protein kinase (MAPK), phosphatidylinositol-3-kinase (PI3K), and protein kinase C (PKC) (Migliaccio et al., 1996; Castoria et al., 2001). Likewise, upon estrogenic stimulation, ERα engages tyrosine kinase receptors as IGF1R, the EGF receptor, and ErbB2 (HER-2/neu), triggering relevant biological effects in diverse cell contexts, including BC cells (Kahlert et al., 2000; Chung et al., 2002; Razandi et al., 2003). For instance, the ERα-mediated activation of growth factor receptors may lead to the stimulation of the Ras/Raf/MAPK and Akt transduction cascades and then to growth responses (Kahlert et al., 2000; Chung et al., 2002; Razandi et al., 2003). Overall, the aforementioned nuclear and extranuclear-initiated pathways driven by ERα may control a variety of biological outcomes in mammary tumor cells, ranging from cell cycle, proliferation, chromatin remodeling to survival, and motility (Ballaré et al., 2003; Levin, 2003; Qiu et al., 2003; Castoria et al., 2004; Vicent et al., 2006; Giretti et al., 2008; Levin and Pietras, 2008; Giovannelli et al., 2012).

Along with ERα, additional mediators have been shown to convey estrogen signaling toward metastatic features. In this regard, the GPER, originally termed GPR30, is a seven-transmembrane protein belonging to the G-protein–coupled receptors superfamily, which mediates the action of estrogens in numerous normal and malignant cell contexts. For instance, several studies have reported a tumor promoting effects of GPER in BC. In this regard, estrogens were shown to trigger through GPER the SRC-mediated extracellular release of heparan-bound EGF and then the activation of EGFR in ER-negative BC cells (Filardo et al., 2000). Triggering rapid kinase-associated transduction pathways (i.e., ERK1/2, PI3K/Akt, Hippo/YAP/TAZ pathway), ion channels (i.e., calcium) and second messengers (i.e., cAMP), GPER may regulate the transcription of diverse genes such as c-fos, connective tissue growth factor (CTGF), EGR1, ATF3, metalloproteases, and cyclins (Pandey et al., 2009; Zhou et al., 2015; Barton et al., 2018). The genomic responses to GPER activation may in turn influence BC cell growth, motility, and invasion (Lappano et al., 2014). Not only estrogens and estrogen-mimetic compounds, but also antiestrogens such as 4-hydroxytamoxifen, raloxifene, and ICI182,780, may act as GPER agonists and stimulate cell survival and proliferative transduction pathways (Filardo et al., 2000; Revankar et al., 2005; Pandey et al., 2009; Prossnitz and Arterburn, 2015). A functional role for GPER in breast tumorigenesis and particularly in metastasis has also been confirmed in transgenic mouse models of mammary tumorigenesis. At later stages of tumorigenesis, GPER knockout mice showed smaller tumors respect to wild-type mice along with a reduced growth rate, histologic features typical of low aggressive tumors, and decreased lung metastases (Marjon et al., 2014). Retrospective BC analysis further supported the contribution of GPER in BC progression. In this vein, immunohistochemical studies showed that GPER levels are positively associated with tumor size, distant metastases, and recurrence in BC specimens and inversely correlated with disease-free survival in tamoxifen-treated patients (Filardo et al., 2006; Liu et al., 2009; Ignatov et al., 2011). A recent bioinformatics analysis in ER-negative BCs has endorsed the aforementioned findings, proving that high GPER levels are both linked with promigratory and metastatic genes and positively correlated with a shorter disease-free interval (Talia et al., 2020). Nevertheless, some studies have reported a tumor suppressor function of GPER (Weißenborn et al., 2014; Martin et al., 2018), warranting further investigations in order to better appreciate the role of GPER in different cancer cell contexts.

### Insulin/IGF Signaling

As stated above, IIGFs, an important growth regulatory pathway often overactivated in BC, is crucially implicated in the acquisition of metastatic features.

IIGFs consists of circulating ligands (insulin, IGF-1, and IGF-2), multiple receptors, and six IGF-binding proteins (Belfiore et al., 2009, 2017). The human IR exists in two isoforms (IR-A and IR-B) generated by alternative splicing of the IR gene with the exclusion (IR-A) or inclusion (IR-B) of 12 amino acids encoded by exon 11. The IR and the IGF-1R have highly homologous structures, but different functions. Given the high degree of homology, IR and IGF-1R can heterodimerize leading to the formation of insulin/IGF-1 hybrid receptors (HRs) (Belfiore et al., 1999, 2009). The IGF-2R lacks an intracellular tyrosine kinase domain and therefore does not transduce intracellular mitogenic signals, acting mainly as a buffer for modulating IGF-2 bioactivity through IR-A and IGF-1R (El-Shewy and Luttrell, 2009). The IIGFs has a significant role not only for normal mammary gland development but also in the onset and maintenance of the malignant phenotype of BC cells. As insulin and IGFs stimulate cell growth via mitogenic, antiapoptotic and chemotactic activity, many of the steps of the normal development of the mammary gland are recapitulated during the process of metastasis (Gallagher and LeRoith, 2011). Indeed, IIGFs is implicated in tumor progression and metastasis of both ER-positive and ER-negative BC cells (Bartella et al., 2012; De Marco et al., 2015) and frequently shows features of deregulation such as (i) overexpression and activation of IGF-1R, IR, and IR/IGF-1R hybrids in malignant cells, (ii) dysregulated expression and/or bioavailability of IGF-1 and IGF-2 in both malignant and stromal cells, and (iii) increased IR-A:IR-B ratio and establishment of IR-A/IGF2 autocrine/paracrine loops (Malaguarnera et al., 2012a). IR-A is also termed the “oncofetal” IR isoform as it exerts a pivotal role in promoting fetal growth by acting as a promiscuous receptor that binds not only insulin but also IGF-2, proinsulin, and IGF-1 (Belfiore and Malaguarnera, 2011; Malaguarnera and Belfiore, 2014; Belfiore et al., 2017). In fact, proinsulin, the insulin prohormone, which is increased in fetal life and insulin resistance conditions, is a high-affinity IR-A ligand (Malaguarnera et al., 2012b) and stimulates proliferation and migration in BC cells.

The increased IR-A:IR-B ratio in BC cells is likely due to multiple mechanisms leading to dysregulated expression of splicing factors involved in exon 11 skipping of the IR gene (Echeverria and Cooper, 2014) including mutations of the gene encoding for the SF3B1 splicing factor. In BC cells IR-A is considered to act as a hub for integrating signals coming from the circulation and connected with the nutritional status (insulin and proinsulin) and signals coming from the microenvironment (IGF-1 and IGF-2) (Belfiore et al., 2017). Insulin has a major orchestrating role in this context by increasing tissue IGFs’ bioavailability through the dual action of enhancing IGF-1 production by the liver and concomitantly inhibiting IGF-BPs synthesis (Belfiore et al., 2017).

IR-A downstream signals show important differences when stimulated by either insulin or IGF-2, the latter being more mitogenic (Frasca et al., 1999). However, IR-A is intimately linked to the mitogenic MAPK/mammalian target of rapamycin (mTOR) cascade rather than to the PI3K/Akt metabolic cascade also in response to insulin (Frasca et al., 1999). As a consequence, BC cells do not share the insulin resistance of peripheral tissues of obese patients (Yee et al., 2020). Therefore, IR-A overexpression can be seen as a way BC cells exploit to overcome insulin resistance of obese patients and allow full response to the estrogen/IIGFs cross-talk (Belfiore et al., 2017). Additionally, IR-A overexpression increases the assembly of IR-A/IGF-1R hybrids that function as high-affinity binding sites for IGFs, thus amplifying signals from the microenvironment (Belfiore et al., 2009). In turn, IR-A–mediated biological responses are regulated by tumor stroma components, such as the proteoglycan decorin, which negatively modulates IGF-2 actions while leaving unaffected insulin/proinsulin effects. Thus, reduced levels of decorin associated with aggressive BCs enhance the activity of the IGF-2/IR-A loop (Morcavallo et al., 2014).

The relevance of this loop is underscored by studies showing that endocrine-resistant ER^+^ BCs may have reduced expression of IGF-1R while expressing much higher levels of IR (Fagan et al., 2012; Yee, 2018). Similarly, data obtained in thyroid cancer indicate that loss of differentiation (Vella et al., 2002) and stem-like phenotype (Malaguarnera et al., 2011) are associated with high relative abundance of IR-A and IGF-2 secretion, while IGF-1R expression is generally reduced.

Although overexpression of IR and IGF-1R in cancer cells recognizes multiple mechanisms, which are reviewed elsewhere (Belfiore et al., 2017), a recently emerged non-mutational mechanism involves the collagen receptor DDR1, which is up-regulated by IIGFs activation and by collagen (Vella et al., 2019a). In turn DDR1 up-regulates both IR and IGF-1R in a feed-forward loop (Matà et al., 2016; Vella et al., 2017) that may enhance BC metastasis potential (see below). Interestingly, DDR1 also regulates adipose cell aromatase and estrogen output by activating a mechanotransduction pathway (Ghosh et al., 2013) representing a relevant node in the estrogen/IIGFs cross-talk.

Not surprisingly, obesity and T2DM, both characterized by insulin resistance, are associated with an increased risk of postmenopausal BC and higher rates of tumor progression and recurrence; hyperinsulinemia has been found to be a major determinant of this risk (Schrauder et al., 2011; Lewitt et al., 2014; Park et al., 2017). In this line, several studies show that women with increased circulating levels of IGF-1 and low amount of IGFBP3 may have a high risk of BC and that high levels of IGF-1 are associated with BC progression and recurrence (Belfiore et al., 2017).

To further corroborate the importance of IR-A activation in BC patients, IR phosphorylation in BC cells was a significant marker of poor patient survival (Law et al., 2008). Moreover, a high IR-A:IR-B ratio was particularly associated with the luminal B subtype of ER^+^/progesterone receptor–positive (PR^+^)/HER2^–^ BCs that are clinically characterized by a higher grade, positive lymph node involvement, and poorer relapse-free survival (Huang et al., 2011).

Notably, the IIGFs is widely implicated in the process of angiogenesis, which is essential for the metastatic dissemination of tumor cells. To metastasize, cancer cells must be able to form new vessels often in hypoxic environments. VEGF-A is an important mediator of angiogenesis and is under the transcriptional control of HIF-1 and HIF-2, transcription factors induced by hypoxia and growth factors (Bielenberg and Zetter, 2015).

Consistently, IGF-1Rs are expressed in isolated hemovascular endothelial cells, newly formed blood microvessels, and in lymphatic endothelium (Bar and Boes, 1984), and IGF-1 is able to up-regulate VEGF through HIF-1α in BC cells. Interestingly, GPER cooperates with HIF-1α for the transcriptional activation of VEGF induced by IGF-1 in vascular endothelial cells (De Francesco et al., 2017). IR-A is also markedly overexpressed in angiogenic vasculature in human tumors and stimulates endothelial cell proliferation and *in vivo* angiogenesis (Belfiore et al., 2009, 2017).

Similarly, lymphangiogenesis is an important mechanism by which tumor cells are disseminated via the lymphatic system and induce lymph node metastases, which occur in the early stages of BC development and may promote further spread of BC cells at distant sites (Fidler, 2003). Both IGF-1 and IGF-2 show the ability to induce angiogenesis and lymphangiogenesis in several *in vitro* and *in vivo* model systems (Bjorndahl et al., 2005). In particular, IGF-1 induces and promotes lymphangiogenesis through the induction of VEGF-C.

Along with angiogenesis and lymphangiogenesis, IIGFs have been implicated in the mechanisms of BC cell homing, which is necessary for colonization at secondary sites. In this regard, several evidences suggest that, upon exposure to cytokines and growth factors of bone microenvironment, BC cells undergo genetic alterations that may enhance their ability to survive and colonize the bone. IGF-1 and IGF-2 are among those molecules found in bone environment together with TGF-β, PDGF, and fibroblast growth factors (FGFs) (Wissmann and Detmar, 2006). Adding to this, oncogene mutations and other molecular abnormalities leading to STAT3 activation induce IGF-2 secretion and IR-A activation toward invasive features and resistance to antitumor treatments (Lee et al., 2006). For instance, IGF-2 secreted by epithelial mammary cells expressing c-Myc oncogene activates fibroblasts that acquire the ability to remodel the ECM, thus promoting epithelial cell invasion (De Vincenzo et al., 2019). Consistently, metastatic BC CAFs have protumorigenic properties induced by increased IGF-2 expression (Gui et al., 2019).

Metabolic reprogramming is a hallmark of cancer. It is worth mentioning that we recently showed that IR-A activation by insulin and IGF-2 plays a role in BC cells metabolic reprogramming by increasing both glycolysis and oxidative phosphorylation. IGF-2–activated IR-A especially enhanced BC cell metabolic flexibility, leading to the acquisition of malignant features consistent with cellular adaptation to a challenging microenvironment characterized by high energy demand (Vella et al., 2019b).

Finally, IGF-2/IR-A loop has also been implicated in EMT (Zelenko et al., 2016) and other stem-like features (Malaguarnera et al., 2011), which play a key role in cancer development and recurrence.

Overall, these studies clearly support a pivotal role for IIGFs in aggressive traits of BC supportive of metastatic phenotypes.

## Microenvironmental Interactions Between Estrogenic Signals and IIGF Conducive to BC Metastasis

**FIGURE 2:**
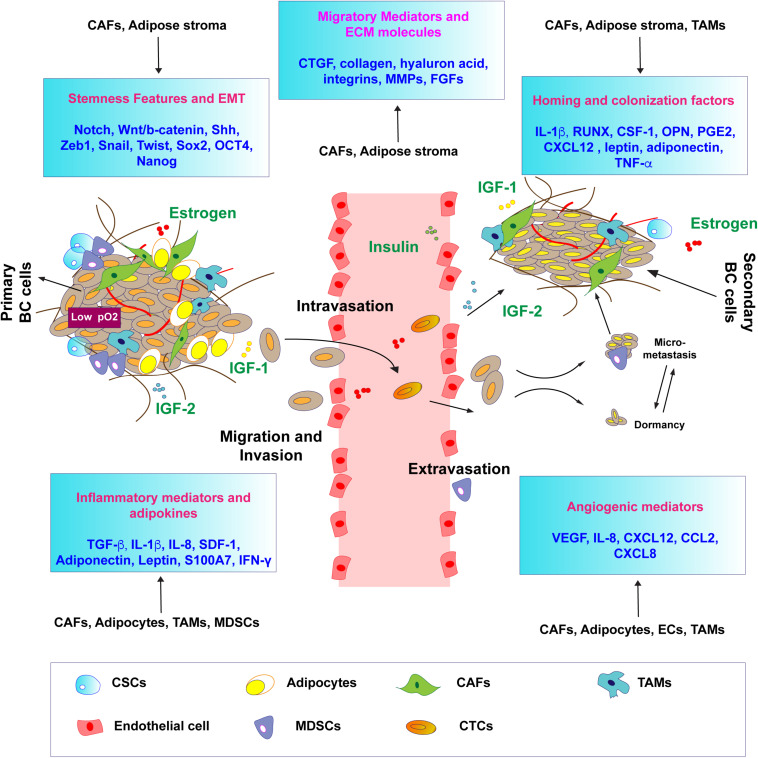
Estrogen and IIGF-prompted microenvironmental responses conducive to BC metastasis. Schematic representation of the main biological responses and shared mediators (in boxes) regulated by both estrogen signaling and by IIGFs, shaping the tumor microenvironment toward metastatic progression. Both estrogen and IIGFs signaling regulate the expression of inflammatory, migratory, and angiogenic mediators by modulating paracrine responses in the tumor microenvironment. The activation of developmental pathways and EMT programs, under the control of estrogen and IIGFs-regulated genes, is responsible for the acquisition of stemness features associated with metastatic progression. Homing and colonization factors under the influence of estrogen and IIGFs trigger BC cells priming to the metastatic sites. CTCs, circulating tumor cell; CSCs, cancer stem cells; CAFs; cancer-associated fibroblasts; TAMs, tumor-associated fibroblasts; MDSCs, myeloid-derived suppressor cells.

As previously mentioned, mounting evidence indicates that, in BC, signals mediated by estrogens and IIGFs shape the tumor microenvironment and drive metastatic evolution. Despite these signaling systems elicit profound direct actions on BC cells themselves, understanding the role of estrogens and IIGFs and their cooperation in landscaping the tumor microenvironment toward metastatic features (Figure 2) may unveil further layers of complexity toward novel therapeutic perspectives. Estrogen/ER-mediated BC progression does involve a bidirectional cooperation between BC cells and components of the surrounding stroma as blood vessels, immune cells, CAFs, and other types of cells (Lappano and Maggiolini, 2018; Rothenberger et al., 2018). Stromal cells may contribute to the progression of BCs acting as a main source of soluble and non-soluble secreted factors such as hormones, growth factors, cytokines, and ECM molecules, which regulate matrix remodeling, neoangiogenesis, migration, and invasion (Lappano et al., 2020a; Tables 1, 2).

**TABLE 1:** Schematic representation of the EMT factors modulated by estrogen and IIGF signaling in BC.

EMT factor	Mediator	Model system	Mechanism involved	References
Snail	IGF-1R	Human mammary epithelial cells	Constitutively activated IGF-IR induces EMT through Snail1	Kim et al., 2007
NF-κB	IGF-1R	Human mammary epithelial cells	Constitutively activated IGF-IR induces EMT through Snail1	Kim et al., 2007
GDF15	IGF-1R	BC cells	GDF-15 activates IGF-1R-FoxM1 signaling to trigger EMT	Peake et al., 2017
TGF-β	IGF-1R	BC cells	IGF-1 and latent TGF-β promote MMPs activity and EMT	Walsh and Damjanovski, 2011
Twist, Zeb, Slug	IR	Immunodeficient hyperinsulinemic mouse models of T2DM and BC cells	Hyperinsulinemia induces IR-mediated EMT	Zelenko et al., 2016
Fibronectin and β-1 integrin	GPER	Tamoxifen-resistant BC cells	GPER/EGFR/ERK signaling upregulates β1-integrin expression and drives EMT	Yuan et al., 2015
IL-1β	E2/GPER	BC cells and CAFs	IL-1β/IL1R1 loop induces EMT	De Marco et al., 2016
Notch, HIF-1α	GPER	BC cells and CAFs	A cross-talk between Notch, HIF-1α, and GPER mediates EMT	De Francesco et al., 2018a
Notch	E2/GPER	BC cells and CAFs	Estrogenic GPER signaling triggers Notch-dependent EMT genes	Pupo et al., 2014
ECM molecules	ERα	BC cells	Loss of ERα triggers EMT	Bouris et al., 2015

**TABLE 2:** Schematic representation of the main stromal mediators involved in metastatic progression by estrogen and IIGF signaling.

Mediator	Regulator	Stromal cell of origin	Target cell/tissue	Metastasis-promoting function	References
Aromatase	Leptin, IL-6	CAFs, adipocytes, ASCs	BC cells and microenvironment	E2 production, cell proliferation, migration, angiogenesis	Luo et al., 2014; Kamat et al., 2015; Sabol et al., 2019
IGF-1 and IGF-2	Oncogenic mutations	CAFs, adipocytes, ASCs	BC cells and microenvironment	Homing, colonization, angiogenesis, EMT, stemness features, CAF activation	Lee et al., 2006, 2016; De Vincenzo et al., 2019
CTGF	E2, IIGFs	CAFs	CAFs, BC cells	Migration, invasion	Madeo and Maggiolini, 2010; De Marco et al., 2013, 2014
Notch	E2	CSCs	CSCs, BC cells	Stemness features, migration, EMT, homing	Pupo et al., 2014
Collagen/DDR1	IIGFs	CAFs	BC cells and microenvironment	Migration, ECM remodeling	Matà et al., 2016; Vella et al., 2017
HIF-1α/VEGF	E2, IGF-1	CAFs	ECs	Angiogenesis	De Francesco et al., 2014, 2017
IL-1β	E2	CAFs	BC cells and microenvironment	Migration, invasion	De Marco et al., 2016
FGF-2	E2	CAFs	BC cells and microenvironment		Santolla et al., 2019
OSM	Adipose stroma	Adipose stroma	CSCs	EMT, stemness features	Lapeire et al., 2014; Sanchez-Infantes et al., 2014; West et al., 2014
PDGF	IGF-1	CAFs	BC cells and microenvironment	EMT, ECM remodeling, intravasation	Pasanisi et al., 2008; Guo and Deng, 2018

In CAFs, the estrogen-induced production of SDF-1α, occurring in an ERα-independent manner, may contribute to BC progression through the accumulation of cancer-infiltrating myeloid-derived suppressor cells (MDSCs) in the tumor microenvironment (Ouyang et al., 2016). In this context, it should be mentioned that growth factors released within the tumor microenvironment may modulate the function of ERα toward the development of breast malignant features (Bartella et al., 2012). Yet, CAFs may be targets of the stimulatory paracrine actions elicited by diverse molecules released by BC and/or other stromal cells (Kalluri, 2016). Among these molecules, IGF-1 and IGF-2 have been shown to be released by epithelial BC cells and drive the acquisition of the activated status in adjacent fibroblasts, toward increased migratory and invasive behavior (De Vincenzo et al., 2019). Conversely, IGF-1 released by CAFs triggered migratory effects in MDA-MB-231 BC cells and the formation of lung metastasis in an animal model of BC (Daubriac et al., 2018). Similarly, the paracrine release of IGF-1 by CAFs primed TNBC to metastasize the bone (Zhang et al., 2013). In parallel, the increased expression of IGF-2 detected in breast CAFs isolated from metastasis, compared to CAFs isolated from primary breast tumors (Gui et al., 2019), suggests that also this growth factor may play a relevant role in the paracrine actions mediated by tumor stroma and leading to the metastatic switch. Indeed, IGFs have been implicated in key stages of bone metastasis such as homing, dormancy, colonization, and expansion (Weilbaecher et al., 2011). In TNBCs, stromal CAFs were identified as the source of IGF-1 and CXCL12, which were shown to prime cells to home the CXCL12- and the IGF1-rich bone microenvironment, in a process dependent on CXCR4 and IGF-1R expression by cancer cells (Zhang et al., 2013). Both IGF-1 and IGF-2 appear to play important roles in bone colonization and expansion by metastasizing tumor cells. In a study, bone-derived IGFs stimulated metastasis of BC to bone by increasing cancer cell proliferation and survival, via AKT activation and recruitment of nuclear factor κB (NF-κB) (Hiraga et al., 2012). Further, culture medium from cells stimulated to undergo bone resorption was found to contain high concentrations of IGF-1; notably, the anchorage-independent growth of human BC cells cultured in this medium was inhibited by the IGF-1R–neutralizing antibody (Ab) αIR3, but not by Abs against TGF-β, FGF-1 or FGF-2, or PDGF-BB (Hiraga et al., 2012). Additionally, growth of human BC cells in a human adult bone model was facilitated by active osteoclasts induced by RANKL, and IGFs released following bone resorption (Sangai et al., 2008). More specifically, CAF-derived IGF-2 triggered migratory effects in BC cells; this effect was elicited through the involvement of the collagen receptor DDR1 (Matà et al., 2016), which has emerged as a pivotal signaling mediator of the IIGFs. In fact, DDR1 not only serves as a receptor for collagen, but it also appears to work as an adaptor signaling molecule necessary for the transduction of IGF-mediated actions (Matà et al., 2016). Interestingly, non-canonical DDR1 signaling was shown to enable collagen action and multiorgan site metastatic reactivation of breast tumors mainly through the activation of the JAK2/STAT3 pathway and the manifestation of CSC traits (Gao et al., 2016). Therefore, collagen-DDR1 signaling may serve as one of the signaling pathways exploited for BC cells’ exit from dormancy, formation of metastasis, and disease relapse. In this context, collagen-enriched ECM integrates hormonal responses toward the establishment of lung metastatic lesions (Jallow et al., 2019). *In vivo*, E2 was able to remodel ECM architecture in the peritumoral area and in the pulmonary premetastatic niche, thus suggesting that both collagen- and estrogen-mediated action may boost lung lesions in ER-positive tumors (Jallow et al., 2019). It should be recalled that the tumor microenvironment at metastatic sites is functionally and molecularly different from the microenvironment surrounding the primary tumor. In particular, a shift from ER-positive to ER-negative context has been detected during metastasis formation. Indeed, Forsare and collaborators interrogated primary and metastatic breast biopsies, as well as CTCs from blood samples serially collected at different timepoints, and demonstrated that the ER status evolves toward the loss of the receptor in CTCs, which reflect real-time tumor progression, as well as at distant metastasis, whereas ER is detectable at the primary tumor site (Forsare et al., 2020). Accordingly, CAFs isolated from primary and metastatic breast tumors were characterized by a differential miRNOma response to estrogens (Vivacqua et al., 2019). These observations suggest that in the microenvironment of breast tumors with aggressive phenotypes, additional mediators may be involved in the stromal response to estrogens. Among these, early studies showed that breast tumor–derived CAFs are stimulated by estrogens through a GPER-mediated nuclear function (Madeo and Maggiolini, 2010; Pupo et al., 2013; Lappano and Maggiolini, 2018). In this regard, GPER, along with the phosphorylated EGFR, was surprisingly recruited by estrogens to the promoter sequences of target genes in CAFs (Madeo and Maggiolini, 2010; Pupo et al., 2013, 2017). Hence, estrogenic GPER signaling fosters CAFs to produce a variety of secreted factors that fuel proliferation, migration, invasion, spreading, and EMT of nearby BC cells, as well as tubulogenesis in endothelial cells (De Francesco et al., 2013; Yuan et al., 2015; De Marco et al., 2016; Pisano et al., 2017; Cirillo et al., 2019; Santolla et al., 2019). In particular, the functional interaction of GPER with the EGFR, IGF1R, FGFR1, HIF-1α, and Notch transduction pathways may trigger the release of growth factors, such as CTGF, VEGF, and FGF2, and cytokines such as IL-1β that account for important paracrine actions mediated by CAFs toward BC growth and dissemination (Pandey et al., 2009; De Francesco et al., 2013, 2014, 2017, 2018a; Pupo et al., 2014; Ren et al., 2015; De Marco et al., 2016; Santolla et al., 2019; Lappano et al., 2020b). Interestingly, diverse studies have shown that GPER bridges together estrogenic signaling with IGF1R and IR-mediated action in the breast tumor microenvironment, independent of the ER status. For instance, the IGF-1/IGF-1R pathway triggers the up-regulation of GPER through the PKCδ/ERK/c-fos/AP1 transduction cascade in an ERα-dependent manner, leading to migratory effects in MCF7 BC cells (De Marco et al., 2013). The cross-talk between IGF-1R and GPER appears to represent a general stimulatory mechanism shared among diverse types of cancer, including mesothelioma and lung cancer (Avino et al., 2016). In addition, IGF-1 stimulation prompted a cross-talk between GPER and DDR-1 leading to cell migration and chemotaxis (Avino et al., 2016). In ER-negative breast CAFs, GPER was shown to be necessary for the stimulatory actions triggered by the metal zinc through the IGF-1R pathway toward CAFs and BC cell migration (Pisano et al., 2017). Furthermore, a functional interaction between GPER and HIF-1α triggered the IGF-1–mediated release of VEGF by CAFs, which prompted vessel-like assembly in endothelial cells. Altogether, these findings suggest that a complex network between ER, GPER, and IGF-1R stimulates the tumor microenvironment and especially CAFs to facilitate metastatic spread. Extending these findings, GPER was shown to be up-regulated not only by IGF-1 but also by insulin in both BC cells and CAFs, thus indicating that GPER may be included among the transduction mediators engaged by the IIGFs pathway in BC (De Marco et al., 2014). The positive correlation between GPER expression in CAFs and serum levels of insulin in BC patients further corroborates the role of insulin in promoting a dysfunctional microenvironment toward disease progression. It should be mentioned that both GPER and the IIGFs have been implicated in the aberrant activation of EMT programs (Table 1), which are known to promote metastasis initiation through multiple mechanisms, such as the gain of stemness properties. In this context, GPER was shown to trigger β1-integrin expression, leading to CAF-induced cell migration and EMT (Yuan et al., 2015). Likewise, estrogenic GPER signaling promoted EMT through the activation of the Notch pathway (Pupo et al., 2014), a signaling system involved in CSC maintenance and survival (De Francesco et al., 2018a). Moreover, in patient-derived xenografts from ER-negative BCs, GPER expression was shown to be higher in breast CSCs compared to the non-CSC counterpart (Chan et al., 2020); phosphoproteomic analysis identified the PKA and BAD-Ser118 as the main transduction mediators involved in GPER signaling in breast CSCs (Chan et al., 2020). Interestingly, GPER silencing reduced CSCs activity *in vitro* and tumor growth *in vivo* (Chan et al., 2020), thus reinforcing the involvement of this receptor in CSC functionality.

Despite the role of estrogenic GPER signaling in regulating breast CSC biology has been recently acknowledged, the contribution of ERs in both normal and CSC remains controversial (Sleeman et al., 2007). Indeed, estrogens appear to rely on receptors others than the classic ERα for the expansion of populations with stem-like features (Fillmore et al., 2010; Alferez et al., 2018). In this context, it should be mentioned that the ER target gene PR plays a key role in the regulation of stemness as evidenced in normal mammary gland development, as well as in the context of breast neoplasia (Daniel and Lange, 2009; Axlund and Sartorius, 2012; Hilton et al., 2012; Finlay-Schultz and Sartorius, 2015; Knutson et al., 2017; Truong et al., 2019).

Likewise, the early dissemination of PR^+^ BC cells has been demonstrated using animal models of BC (Hosseini et al., 2016). Extending these findings, PR signaling has been shown to synergize with ER pathway to regulate a number of effectors involved in stemness, metastatic proficiency, and resistance to therapy (Hilton et al., 2012; Finlay-Schultz and Sartorius, 2015; Mohammed et al., 2015; Diep et al., 2016). Among these mediators, the PR target gene insulin receptor substrate 1 (IRS-1), which is a relevant member of the IIGFs, may represent a novel node, bridging together ER-signaling and IIGF signaling by means of PR (Daniel and Lange, 2009).

As it concerns the IGF system, IGF-1R represents a very well-known driver of EMT and stem-related functions in normal and cancerous tissues. Stem-promoting signaling pathways such as Notch, Wnt/β-catenin and Shh may function upstream of IGF-1R to increase its expression (reviewed in Farabaugh et al., 2015): in addition, signaling cascades downstream of IGF-1R activate transcription factors involved in the control of EMT and stemness, such as Zeb1, NF-κB, Snail, Twist, Sox2, Oct4, Nanog (reviewed in Farabaugh et al., 2015). It has been reported that IGF-1 signaling has a critical role in BC progression by controlling both the maintenance of BCSCs and their EMT behavior (Chang et al., 2013). However, IGF-1 can enable EMT also through the activation of non-classical EMT factors; this is the case for transmembrane glycoprotein MUC1, which is frequently overexpressed in BC metastasis, and is up-regulated through the IGF-1R/PI3K/AKT pathway (Cordone et al., 2017). Intriguingly, targeting MUC1 may reverse BC stem cell phenotype, thereby supporting the role of MUC1 in metastatic dissemination. The mammary tissue is rich in adipocytes that produce multiple endocrine, inflammatory, and angiogenic factors involved in the growth and the acquisition of malignant and stem cell traits by adjacent breast tumor cells (Lee et al., 2015). Accordingly, a number of experimental evidences have supported the role of adipocytes in the establishment of metastasis in BC (Kamat et al., 2015). As mentioned above, estrogen production in adipocytes could be one of the mechanisms involved in the higher incidence and aggressiveness of BC observed in obese postmenopausal women. It is been demonstrated that aromatase activity in differentiated adipocytes, as well as in adipose stem cells, is fostered by the hormone leptin, as well as by other adipokines such as IL-6, with the result to increase local estrogen production and ERα signaling (Liu et al., 2013; Strong et al., 2013; Sabol et al., 2019). Beyond estrogen production, other obesity-related factors can contribute to the acquisition of metastatic phenotypes in BC patients. For instance, obesity is associated with a low-grade chronic inflammatory state, characterized by increased production of inflammatory mediators, together with enhanced IGF-1 and insulin signaling (Iyengar et al., 2013). In this context, it should be mentioned that inflammatory factors produced by adipose cells subjected to fat overload contribute not only to insulin resistance, but also to increased metastatic propensity. In conditions of obesity, the adipose tissue is highly inflammogenic as the stressed adipocytes undergo hypoxia and eventually death, thereby liberating several signaling molecules from dying cells. These damage-associated molecular patterns in turn attract immune cells such as macrophages, which enwrap dying adipocytes to form crown-like structures and foam cells. Inflammatory cytokines such as TNF-α, IFN-γ, IL-1β, IL-6, and HMGB1 are released either by adipocytes or activated macrophages to recall additional immune cells and perpetuate the inflammatory damage. Certain inflammatory mediators secreted from the adipose tissue of breast tumors have been shown to trigger direct stimulatory effects on BC cells. For instance, the migration rate of BC cells was increased after coculture with carcinoma adipose stromal cells; this effect was shown to be dependent on the up-regulation of the small calcium binding protein and inflammatory mediator named S100A7, which is correlated with adverse pathological parameters and poor relapse-free survival (Sakurai et al., 2017). Likewise, oncostatin M (OSM) and other adipokines released from tumor-associated adipose tissue prompted the activation of STAT3, and its target genes S100A7, S100A8, and S100A9 triggering increased cellular scattering and peritumoral neovascularization of orthotopic xenografts (West and Watson, 2010; Lapeire et al., 2014). Adding to this, cytokines such as IL-6, IL-1β, and TNF-α, as well as adipokines such as leptin and adiponectin released by bone marrow adipocytes, send homing signals for BC cells to colonize the bone tissue (Choi et al., 2018a). Thereafter, the process of metastasis priming at distant site can be facilitated by a number of adipocyte-derived paracrine factors whose expression is often regulated by both estrogens and IIGFs. This is the case for IL-1β, which is a transcriptional target of signals mediated by GPER (De Marco et al., 2016), ER (Ruh et al., 1998), and IGF-1R (Ho et al., 2017) toward increased invasiveness and metastatic aggressiveness (De Marco et al., 2016; Eyre et al., 2019). In addition, IL-1β is involved in the activation of obesity-induced insulin resistance and inflammation. In fact, reduced gene expression, protein abundance of insulin signaling molecules, and increased release of inflammatory mediators were observed in adipocytes stimulated with IL-1β (Gao et al., 2014). Furthermore, IL1-β was shown to promote stem-cell–like phenotypes and invasiveness in MCF7 BC cell through the up-regulation of IL-6 (Oh et al., 2016), which has been shown to be released not only by cancer cells but also by adipocytes, CAFs, and TAMs (Gyamfi et al., 2018; Xu et al., 2019). Beyond the ability to promote the release of proinflammatory molecules such as IL-1β and IL-6 in the tumor microenvironment, estrogens and IIGFs-mediated signals have been shown to cross-communicate with certain adipokines such as leptin. As mentioned previously, leptin increases the availability of estrogens and promotes migration, invasion, EMT, and CSC enrichment in BC (Strong et al., 2015). A well-documented cross-talk between leptin and IGF-1R signaling pathways has been shown to promote the migration and invasion of BC cells (Saxena et al., 2008). Furthermore, leptin pathway cooperates with ER-mediated signaling to trigger stimulatory actions in BC (Fusco et al., 2010).

TAMs may comprise up to 50% of the BC microenvironment (Obeid et al., 2013). TAMs regulate the secretion of growth factors, proinflammatory cytokines and chemokines leading to the resistance to endocrine therapy, tissue remodeling, angiogenesis, suppression of immune responses, and tumor growth (Obeid et al., 2013). Consequently, TAMs are associated with an increased aggressiveness and worse outcomes in breast malignancy (Williams et al., 2016; Choi et al., 2018b). For instance, TAMs may induce tamoxifen resistance through the activation of the PI3K/Akt/mTOR transduction pathway in BC cells (Li et al., 2020). Likewise, macrophage differentiation in TAMs mediated by the Notch signaling may promote BC resistance to the aromatase inhibitors (Liu et al., 2017). Interestingly, BC cells exposed to conditioned medium from TAMs have been shown to exhibit loss of ERα expression, increase of the proliferative marker Ki67, and the activation of c-Src, PKC, and MAPK transduction pathways, further supporting a role for TAMs in the endocrine resistance and BC patients’ prognosis (Stossi et al., 2012). Together with CAFs, TAMs are the main source of IGFs within both primary and metastatic tumors. High macrophage infiltration has been associated with a poor prognosis and increased rates of metastasis in several cancer types, as TAMs can facilitate blood vessel formation to support expanding tumor growth and aid tumor cell intravasation into vasculature (Chittezhath et al., 2014). Soluble factors present in the TME, such as IGFs, may recruit and influence macrophage behavior (Hao et al., 2012). For instance, macrophages have been shown to play a role in matrix organization through the secretion of MMPs that are capable to degrade and reorganize the matrix, as well as aid in tumor cell migration (Kessenbrock et al., 2010). Moreover, TAMs have been shown to facilitate the deposition of aligned collagen fibers during tumor development (Varol, 2019). The binding of these ECM proteins to adhesion receptors on the surface of macrophages promotes inflammatory and tumor-promoting macrophage activation (Hsieh et al., 2017). Alterations in ECM organization and composition in the tumor microenvironment result in increased matrix stiffness, primarily localized at the invasive front of breast tumors. These stiff regions are enriched in aligned collagen fibers and TAMs. Studies have demonstrated that substrate stiffness, which is associated with enhanced breast tumor progression, is another mechanical aspect of the ECM that can influence macrophage behavior. Matrix stiffness, increasing CCL2 levels, may recruit specific macrophage populations, which interact with collagen fibers and facilitate tumor cell dissemination. Thus, it is becoming clear that macrophages are sensitive to changes in the ECM and their mechanical environment. In agreement, activation of IIGFs in BC patients has been correlated with increased macrophage infiltration, advanced tumor stage, resistance to therapies, and poor prognosis (Campbell et al., 2011). Stroma-derived IGFs have been further investigated in BC progression and metastasis, and the therapeutic opportunity of blocking IIGFs in combination with chemotherapy has been also evaluated. For instance, the efficacy of paclitaxel, a chemotherapeutic agent commonly used for the treatment of invasive BC, has been shown to be increased by the concomitant block of IGFs (Ireland et al., 2018). Altogether, these findings indicate that estrogens and IIGFs may cooperate to elicit a multifaceted breast tumor–supporting action through CAFs, tumor-associated adipocytes and macrophages, and other important components of the tumor stroma. By shaping relevant paracrine interactions within the tumor microenvironment, estrogen and IIGFs signaling systems may play a key role in the development and progression of BC metastasis.

## Manipulating the Cross-Talk Between Estrogenic Signals and IIGF to Halt Metastatic Progression

Hormone therapy targeting the ER-mediated pathway is largely used for ER-positive breast tumors, which account for approximately 75% of all BCs (Senkus et al., 2013). Despite the good outcome, certain ER-positive tumors may become resistant to treatments and relapse, leading to a poor prognosis (Osborne and Schiff, 2011; Ma C.X. et al., 2015). Multiple mechanisms responsible for the endocrine resistance have been proposed including the activation of escape pathways toward alternate proliferative and survival stimuli (Osborne and Schiff, 2011; Ma C.X. et al., 2015). In this vein, diverse BC subtypes commonly express high levels of main players of IIGFs (Bhargava et al., 2011; Bahhnassy et al., 2015). Therefore, targeting IIGFs has been suggested as a promising therapeutic approach in BCs (Christopoulos et al., 2018). Accordingly, many components of the IGFs have been indicated as suitable targets on the basis of the results obtained in preclinical studies (Motallebnezhad et al., 2016). Unfortunately, clinical trials, particularly phase III studies, performed in BC patients, provided rather disappointing data for the rise of adverse effects together with minimal clinical benefit (Philippou et al., 2017; Qu et al., 2017). Hence, strategies cotargeting the bidirectional network between the estrogen and IIGFs could be exploited toward successful treatments (Table 3). In this regard, in a clinical trial for advanced ER-positive BCs, the use of the IGF-1R Ab figitumumab combined with the aromatase inhibitor exemestane has provided encouraging results in patients without preexisting metabolic syndrome at the time of the enrollment (Ryan et al., 2011). On the contrary, the addition of the IGF-1R therapeutic monoclonal Ab ganitumab to exemestane or fulvestrant did not improve the outcomes (Robertson et al., 2013). Moreover, experimental findings indicating an increased ratio of IR-A:IR-B in ER-positive BCs (luminal B) have suggested that targeting both IR-A and IGF-1R, along with the estrogen signaling, may be beneficial in these patients, therefore avoiding the compensatory cross-talk between IGF-1R and IR (Huang et al., 2011; Yee, 2012). In this regard, a phase II study (NCT01205685) investigated in ER-positive BC patients the potential antitumor activity of a dual IGF-1R/IR tyrosine kinase inhibitor, namely, linsitinib (OSI-906), used in combination with hormone therapy. Unfortunately, this study was ended because of the appearance of severe toxicities associated with the treatments. To date, much focus has been turned into the design of novel molecules showing an enhanced efficacy without adverse effects and the identification of natural compounds able to trigger the desired action. Picropodophyllotoxin (PPT) is an epimer of podophyllotoxin isolated from the roots of *Podophyllum hexandrum*, which has been used as an antitumor drug and insecticidal/antifungal agent (Liu et al., 2015; Zhi et al., 2017). Launched as an anticancer drug targeting specifically the IGF-1R autophosphorylation (Girnita et al., 2004), PPT was shown to prevent the paracrine recruitment of fibroblasts and their activation as CAFs by breast tumor cells expressing c-Myc (De Vincenzo et al., 2019). PPT was also evidenced to suppress the capacity of CD24^–^CD44^+^ BC stem cells to undergo the EMT process (Chang et al., 2013). Promising experimental data have been provided using a dual IGF-1R/IR tyrosine kinase inhibitor, named BMS-536924, which showed the capability to prevent proliferative and migratory features of BC cells (Law et al., 2008; Litzenburger et al., 2009), without adverse effects associated with the insulin deficiency (Dool et al., 2011). Furthermore, a tyrosine kinase inhibitor targeting both IGF-1R and IR, named BMS-754807, triggered an inhibitory response in TNBC cells characterized by an IGF signature (Litzenburger et al., 2011). Likewise, TNBC cells derived from mice inoculated with both cancer cells and mesenchymal stem cells exhibited a reduced formation of bone metastasis using the BMS-754807 (Zhang et al., 2013). Unfortunately, clinical evidence regarding the action of both BMS-536924 and BMS-754807 in breast tumors, either using each inhibitor alone or in combination with hormone therapeutics, is still lacking. The interaction of tumor cells with the surrounding stroma profoundly influences the etiology and progression of BC through multiple mediators including hormones, growth factors, and cytokines. For instance, tumor–stroma communications may provide within the breast microenvironment growth factors such as IGFs, which in turn activate the ER-mediated signaling (Bartella et al., 2012). Similarly, the alternate ER GPER interacts with the IGF-1R transduction pathways acting as a mediator of the multifaceted estrogen action on breast CAFs (De Marco et al., 2013, 2014, 2016; Lappano et al., 2013; De Francesco et al., 2017; Pisano et al., 2017). Together, novel therapeutic approaches targeting the tumor–stroma network are required in order to inhibit the various molecules secreted within the tumor microenvironment and the downstream pathways prompting the proliferation, invasion, and resistance to chemotherapy of the tumor cells. In this context, size-switchable nanoparticles that deliver chemotherapeutics and simultaneously halt the stimulatory action of important regulators of the cancer microenvironment have been proposed in order to improve the treatment outcomes (Cun et al., 2019). As a therapeutic option in BC, an approach targeting downstream effectors of the cross-talk occurring between estrogen and IIGFs has been also suggested. Among others, valuable candidates are the inhibitors of the PI3K pathway (Jia et al., 2008), which is mainly involved in the IGF-1R–mediated action (Ciruelos Gil, 2014; Kasprzak et al., 2017). Moreover, a cross-talk between the PI3K/AKT/mTOR and the ER transduction cascades may occur either directly or through the IGF-1R effector, namely, IRS-1 (Guvakova and Surmacz, 1997; Ciruelos Gil, 2014). Hence, this latter mediator could be considered as a further potential target of the estrogen and IGFs network in BC. Indeed, IRSs are adapter proteins that interact with both IR and IGF-1R toward the stimulation of cell growth, motility, and metastasis (Pirola et al., 2003; Becker et al., 2016). Serving as scaffolds in BC cells, IRSs activate other intermediate proteins including the PI3K/AKT/mTOR signaling (Law et al., 2008; Mirdamadi et al., 2015). Of note, estrogens trigger the up-regulation of IRS-1 activating the PI3K transduction pathway (Guvakova and Surmacz, 1997; Sisci et al., 2007). Accordingly, the silencing of IRS-1 enhanced the tamoxifen-induced cell death in BC cells (Cesarone et al., 2006) and abrogated the transcriptional activity of ER dependent by IGF-1 (Sisci et al., 2007).

**TABLE 3:** Main combination therapies targeting the IIGFs and estrogen signaling.

Combination therapies targeting		References or ClinicalTrials.gov identifiers
IIGFs	Estrogens	
Figitumumab	Exemestane	Ryan et al., 2011
Ganitumab	Exemestane or fulvestrant	Robertson et al., 2013
Linsitinib	Letrozole	NCT01205685
MEDI-573	Letrozole, anastrozole, or exemestane	NCT01446159

Because of the multilevel paracrine actions elicited by both IGF-1 and IGF-2 in BC metastasis, it is plausible to hypothesize that the direct targeting of IGF-1 and/or IGF-2 would provide an interesting strategy in therapeutic setting. The ligand-neutralizing approach has been tested in preclinical and clinical studies in diverse types of solid tumors, including BC. For instance, the neutralizing human Ab MEDI-573 serves as a double inhibitor for IGF-1 and IGF-2. In animal models, MEDI-573 blocks tumor growth by halting the IGF-1R and IR-A signaling cascade (Iguchi et al., 2015). Because of the encouraging results, MEDI-573 is currently under investigation in a phase 1b/2 clinical trial in patients with metastatic HR^+^/HER2^–^ BC, in combination with aromatase inhibitors (NCT01446159). Preliminary data have shown that MEDI-573 suppresses IGF-1 and IGF-2 without generating dose-limiting toxicity including metabolic disorders (Iguchi et al., 2015). The monoclonal Ab neutralizing IGF-1 and IGF-2 named BI836845 is also being tested in a cohort of HR^+^/HER2^–^ metastatic BC patients, in combination with mTOR and aromatase inhibitors, in a phase 2 clinical trial (NCT02123823). Furthermore, Vaniotis et al. (2018) generated a soluble fusion protein consisting of the extracellular domain of human IGF-1R and the Fc domain of human IgG. This product, named IGF-TRAP, showed IGF-1 and IGF-2–binding activity with elevated affinity, which was threefold higher than that of insulin (Vaniotis et al., 2018). The IGF-TRAP exhibited potent anti-antimetastatic bioactivity in BC, thus representing a novel tool for better manipulation of metastatic disease (Vaniotis et al., 2018).

Strategies cotargeting both estrogen and the IGF signaling as well as the cross-communication with protumorigenic molecules such as the adipokine leptin or the proinflammatory cytokine IL1-β would appear to offer major beneficial effects with respect to the inhibition of a single signaling pathway. In this vein, it should be mentioned that leptin inhibition reversed the breast CSC phenotype (Giordano et al., 2016), as well as lessened the effects exerted by adipose-derived stem cells (ASCs) derived from obese BC patients on cancer cell growth (Strong et al., 2013). Approved by the US Food and Drug Administration for the treatment of rheumatoid arthritis, the IL-1 antagonist anakinra showed in BC a remarkable safety record together with a suppressive action on the IL-1–related inflammatory effects (Wu et al., 2018). To date, a single pilot trial aimed at determining the safety of anakinra used along with chemotherapy in patients with metastatic BCs is currently undergoing (NCT01802970). Overall, these findings may suggest that investigating the potential of combination strategies might provide further cues and clinical advantages in BC patients.

## Discussion

Metastatic BCs continue to be a foremost challenge as they are almost always incurable, ultimately leading to death (DeSantis et al., 2019). The poor clinical prognosis is further exacerbated by the lack of effective targeted treatments and by acquired resistance to therapies. Notwithstanding the advances made with targeted therapies, the absence of defined molecular targets and the high tumor heterogeneity of metastatic BC have resulted in lack of benefit in several subgroups of these patients (Mutebi et al., 2020). The discovery of new molecular targeting agents for metastatic BC is therefore an unmet need. Metastatic disease and therapy resistance are highly correlated with intracellular activated pathways. While previous studies have been mainly focused on genetic and biological differences between primary and metastatic epithelial BC cells, more recently, attention has gradually shifted to the most important cellular components of tumor stroma ascribing an increasing importance to cells of tumor microenvironment (Hanahan and Coussens, 2012; Guo and Deng, 2018). During cancer progression, both malignant epithelial and stromal cells produce various components and/or remodelers of ECM that promote metastatic progression, establishing the concept that tumor microenvironment has an essential role in BC biology and therapeutic response (Hanahan and Coussens, 2012; Guo and Deng, 2018). Extensive differences in tumor stroma compared with normal stroma have been widely observed, and several studies have shown that tumor microenvironment may affect biology and progression of cancer cells influencing therapeutic response and clinical outcome (Cacho-Díaz et al., 2020). Differences in tumor microenvironment of primary tumor and metastatic lesions have been reported. For instance, tumor cells are more protected in metastatic lesions than in primary tumor by tumor microenvironment (Cacho-Díaz et al., 2020). Soluble factors secreted by tumor or stromal cells, as well as ligand–receptor interactions and downstream pathways activation, play a pivotal role. Thus, we can expect that the full comprehension of underneath defects could be precious in future therapeutic perspectives.

The importance of IIGFs and estrogenic signaling in BC is well-established, as is the cross-talk between these pathways. However, relatively little is known regarding the impact of this cross-talk in modulating BC cells/microenvironment interactions, especially regarding BC metastatic evolution. We have focused on evidence showing that, indeed, estrogen/IIGFs impacts on stroma at different levels and that, conversely, tumor stroma itself is a main source of soluble and non-soluble secreted molecules, which regulate ECM remodeling, neoangiogenesis, migration, and invasion. In particular, dysregulated expression and bioavailability of IGFs have been implicated in key stages of metastasis, while estrogenic signaling toward the development of breast malignant features (Bartella et al., 2012; De Marco et al., 2015). Noteworthy, estrogen production by adipocytes has been linked to the higher incidence and aggressiveness of BC in obese postmenopausal women (Park et al., 2017).

Hopefully, a better knowledge of the impact of the estrogen/IIGFs cross-talk in modulating BC metastasis by affecting tumor microenvironment could have translational implications. Interestingly, IIGFs is regulated by ER but becomes the reliant signaling pathway when the expression and activation of ER are lowered by long-term blockade of ER signaling. In parallel, GPER signaling, which contributes to tamoxifen resistance, is crucially involved in a bidirectional cross-talk with IIGFs (Bartella et al., 2012; De Marco et al., 2015).

As already mentioned, IIGFs and estrogen signaling pathways are molecularly interconnected and result in redundancies and compensations that contribute to BC aggressiveness. Consistently, IIGFs inhibition have been exploited to overcome BC resistance and improve clinical outcome; however, an ideal way to inhibit IGF-1R, IR-A, and hybrid IR-A in cancer is still lacking. To date, several potential strategies against IIGFs and estrogen system activation have been attempted, but targeting a single system has failed to improve clinical outcome. Definitely, we propose that a combined approach strategy is mandatory.

In summary, we believe that targeting the tumor–environment interaction by focusing on the estrogen–IIGFs cross-talk may represent an effective therapeutic option, especially in patients with hyperinsulinemia due to insulin resistance. However, further studies are still needed to explore this challenging therapeutic option.

## Author Contributions

VV, ED, and RL collected and discussed the literature and wrote the manuscript, in collaboration with MGM and LM. AB and MM coordinated the work, and substantially integrated the manuscript, which was revised and approved, in its final version, by all authors. All authors contributed to the conceptualization and design of the review.

## Conflict of Interest

The authors declare that the research was conducted in the absence of any commercial or financial relationships that could be construed as a potential conflict of interest.
